# Development and application of high-resolution melting analysis for the classification of infectious laryngotracheitis virus strains and detection of recombinant progeny

**DOI:** 10.1007/s00705-018-4086-1

**Published:** 2018-11-12

**Authors:** Omid Fakhri, Carol A. Hartley, Joanne M. Devlin, Glenn F. Browning, Amir H. Noormohammadi, Sang-Won Lee

**Affiliations:** 1 Asia-Pacific Centre for Animal Health, Faculty of Veterinary and Agricultural Sciences, The University of Melbourne, Parkville, VIC Australia; 2 Asia-Pacific Centre for Animal Health, Faculty of Veterinary and Agricultural Sciences, The University of Melbourne, Werribee, VIC Australia; 3 College of Veterinary Medicine, Konkuk University, Seoul, Republic of Korea

## Abstract

Live attenuated vaccines against infectious laryngotracheitis virus (ILTV) are widely used in the poultry industry to control disease and help prevent economic losses. Molecular epidemiological studies of currently circulating strains of ILTV within poultry flocks in Australia have demonstrated the presence of highly virulent viruses generated by genomic recombination events between vaccine strains. In this study, high-resolution melting (HRM) analysis was used to develop a tool to classify ILTV isolates and to investigate ILTV recombination. The assay was applied to plaque-purified progeny viruses generated after co-infection of chicken embryo kidney (CEK) monolayers with the A20 and Serva ILT vaccine strains and also to viruses isolated from field samples. The results showed that the HRM analysis is a suitable tool for the classification of ILTV isolates and can be used to detect recombination between ILTV vaccine strains *in vitro*. This method can be used to classify a broad range of ILTV strains to facilitate the classification and genotyping of ILTV and help to further understand recombination in these viruses.

**Electronic supplementary material:**

The online version of this article (10.1007/s00705-018-4086-1) contains supplementary material, which is available to authorized users.

## Introduction

Infectious laryngotracheitis (ILT), caused by infectious laryngotracheitis virus (ILTV, species *Gallid alphaherpesvirus 1*), has an impact on the poultry industry worldwide [1]. Live attenuated vaccines are widely available and are often administered in the early life of commercial birds, followed by one or more rounds of revaccination in order to reach the desired level of immunity [2, 3]. Although the level of immunity developed by this vaccination procedure is adequate to protect the chickens from developing clinical disease (respiratory signs followed by reduced weight gain and egg production), the challenge virus is often able to replicate in the vaccinated birds [2, 4].

All of the ILTV strains form a single serotype [5], yet have shown a notable level of genotypic variation in different studies (0.1–0.8% nucleotide sequence difference) [6]. A number of different methods have been published to classify these viruses by genotype [7–10], including polymerase chain reaction restriction fragment length polymorphism (PCR-RFLP) [9–13], targeted sequencing [7, 8], and strain-specific fluorescent probe hydrolysis [14]. In all methods, multiple regions of the ~150-kbp genome are analyzed in order to more precisely differentiate genotypes. These methods have been used to classify ILT viruses to facilitate epidemiological studies and clinical diagnosis [15].

Intraspecific genomic recombination is considered an important driver in the evolution of ILTV and other herpesviruses [16] that have low synonymous nucleotide sequence substitution rates [17]. Genomic recombination requires coinfection with two or more strains, where replication of challenge virus in vaccinated chickens and reactivation of latent ILTV infection (either vaccine or field strains) can provide an environment that allows genomic recombination [18]. Intraspecific genomic recombination between vaccine strains of ILTV has been reported to occur in the field [18] and has been demonstrated for field strains under laboratory conditions [14, 18–20]. However, the recombination between modified live vaccine strains has not been studied in experimental settings.

High-resolution melting (HRM) analysis is a simple, rapid and cost-efficient method for genotyping of multiple single-nucleotide polymorphisms (SNPs) [21]. This method has been used in several pathogen identification procedures [22], as well as mutation scanning and diagnosis of genetic disorders in human genome research [23–25]. The extensive applications of this method demonstrate its wide potential to be used as an adjunct to current detection methods.

This study aims to develop an accurate and cost-effective HRM analysis that can be applied to the classification of known and emerging ILTV strains, and to study genomic recombination of ILTV vaccine strains under controlled *in vitro* conditions.

## Materials and methods

### Viruses

The eight ILTV isolates used in this study are listed in Table 1. The viruses (other than the commercial vaccine viruses) were plaque purified, propagated on primary CEK cells and re-typed by PCR-RFLP [9] prior to testing. In order to increase the titre of commercial vaccines, each vaccine was passaged twice on the chorioallantoic membranes (CAM) of specific-pathogen-free (SPF) embryonated eggs (Australian SPF Services, Woodend, Australia) using artificial air-sac inoculation [26]. The infected CAMs were extracted and thoroughly minced using a mortar and pestle. The homogenates were allowed to sediment for 5 minutes at room temperature to let the larger fragments settle before the supernatants were collected. The harvested viruses were cultured on CEK monolayers. Viruses were harvested at 48 hours after infection of monolayers, and aliquots were stored at -80 °C.

**Table 1:** Viruses used in this study

Isolate	PCR-RFLP class [9]	GenBank accession number	Reference
NOBILIS^®^ ILT (Serva)	7	HQ630064	MSD Animal Health
Poulvac^®^ Laryngo A20	1	JN596963	Zoetis
ACC78	8	JN804826	[18]
Class 9	9	JN804827	[18]
Class 10	10	KR822401	[15]
SA2	1	JN596962	[6]
CSW-1	4	JX646899	[19]
V1-99	2	JX646898	[19]

### Cell culture

CEK cells were prepared using kidneys harvested from 18-day-old chick embryos as described previously [27]. Monolayers of CEK cells were maintained in Dulbecco’s modified Eagle medium (DMEM) containing 1% v/v foetal bovine serum (FBS), and 100 μg of ampicillin and 10 μg of amphotericin B per mL. All *in vitro* and *in ovo* experiments were performed on CEK cells or CAM from embryonated SPF hen eggs (Australian SPF Services, Woodend, Australia).

### Coinfection of CEK monolayers

Primary CEK monolayers were infected with a mixture of Serva and A20 ILTV strains in 6-well tissue culture plates. After 1 hour of incubation at 37 °C, the inoculum was removed and 3 mL of fresh medium was added. The total multiplicity of infection (MOI) for the coinfection studies was 10. The progeny virus populations were collected by scraping the cells into culture supernatant 48 hours post infection when an extensive cytopathic effect was observed. Samples collected from this experiment were stored at -80 °C prior to virus isolation and plaque purification.

### Virus isolation and plaque purification

Ten-fold dilutions of progeny viruses from an *in vitro* coinfection experiment were prepared and inoculated onto CEK monolayers in 6-well plates as described previously [14]. Following 1 hour of incubation at 37 °C, the inoculum was removed and the monolayers were overlaid with methylcellulose semi-solid medium containing 1% w/v methylcellulose in DMEM, with 1% v/v FBS, and 50 μg of ampicillin, 50 μg of gentamicin and 5 μg of amphotericin B per mL. After 48 to 72 hours of incubation at 37 °C in a humidified atmosphere of 5% v/v CO_2_, 20 well-isolated plaques were picked using a micropipette guided under an inverted light microscope. Picked plaques were resuspended in 500 μL of DMEM, and each plaque was purified through three rounds of plaque purification before a final propagation step on CEK cells in 24-well plates. One freeze/thaw cycle was performed between rounds of plaque purification.

### DNA extraction

Nucleic acid extraction was performed using a PureLink^®^ Pro 96 viral RNA/DNA purification kit (Invitrogen, USA) and a QIAxtractor automated vacuum system. Purified DNA from 200-μL samples was eluted in 200 μL of pyrogen-free water (Milli-Q^®^ Integral system, Darmstadt, Germany) and stored at -20 °C until testing. Pyrogen-free water, rather than the elution buffer supplied with the extraction kit, was used to avoid introducing any variation in HRM patterns.

### HRM-based multiple-SNP ILTV genotyping assay

#### Assay conditions

**Fig. 1:**
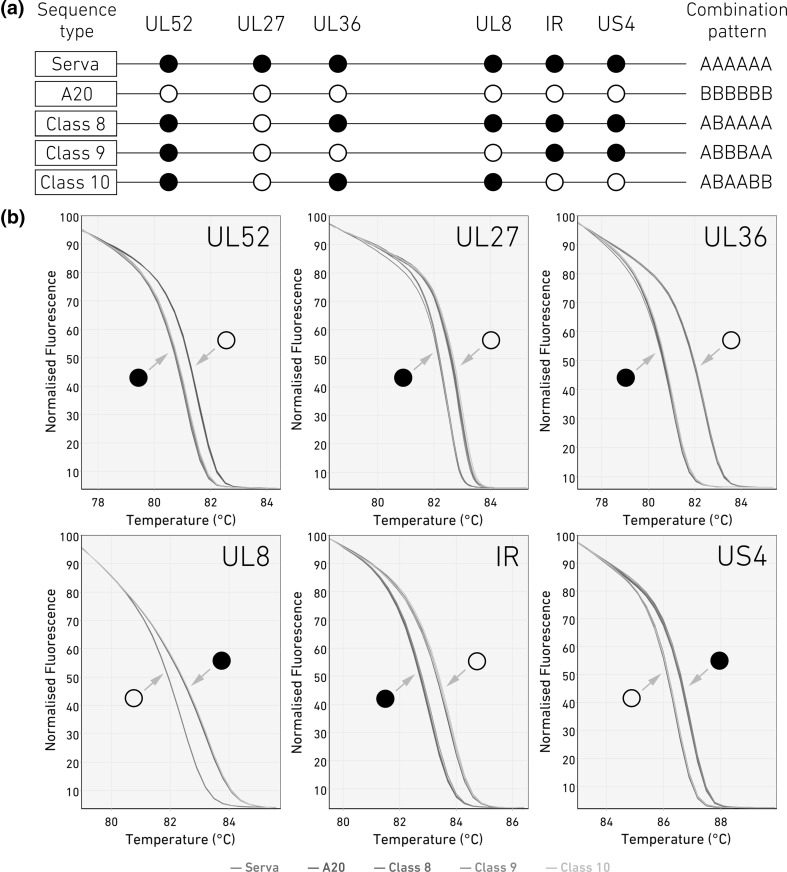
HRM analysis of five fully sequenced ILTV isolates. (a) A schematic view of six target genomic markers with distinct melting characteristics showing the distribution of Serva-like (closed circles) and A20-like (open circles) SNPs in Serva, A20, class 8, 9 and 10 ILTV strains. (b) HRM plots showing the differentiation of Serva (red) and A20 (blue), along with the melt curves for class 8 (green), class 9 (orange) and class 10 (purple) ILTV strains by the six target genomic markers (UL52, UL27, UL36, UL8, IR and US4). Each sample was clustered into SNP identities that were either Serva-like (closed circles) or A20-like (open circles). The plots were generated by Rotor-Gene Q software. Each curve represents one replicate

HRM assays were developed to be used as a method of classification and genotyping ILTV. Following the alignment of full genome sequences of the virus isolates (Table 1), 662 SNPs were identified. These potential target regions were further analyzed *in silico* using uMelt software [28] in order to identify the regions with a detectable melting temperature (*T*_m_) gap in their PCR products. Ultimately, six regions were selected based on their even distribution across the genome (in order to increase the chance of detecting recombinants), the ability to measure differences in their *T*_m_ values, and their ability to produce unique patterns for known/tested ILTV strains. Targeted sites included both transition and transversion mutations and resulted in either synonymous or non-synonymous changes (Table 2 and 3). The primers used for this assay are listed in Table 2. A unique single-nucleotide polymorphism (SNP) pattern was predicted for each strain based on the SNPs present in six genomic regions (Fig. 1A). These regions were also amplified and sequenced (Big Dye Terminator v3.1, Life Technologies) to confirm the presence of the targeted SNPs in these viruses.

**Table 2:** List of primers and selected target SNPs tested on Australian ILTV isolates

Target gene	Primer set (5’-3’)	Annealing temp. (°C)	Amplicon length	*T*_m_ gap (°C) experimental [predicted^a^]	No. of SNPs in amplicon	Type of SNP (NS/S)^b^	SNPs							
							Serva	A20	CL8	CL9	CL10	SA2	CSW-1	V1-99
UL52	F: GGTCCGTTTCTAGCTGTTGG R: TTGCTCCCTTAAGTAGGTTACTTT	49	130	0.4 [0.8]	2	TS (S) TV (NS)	A T	G G	A T	A T	A T	G G	G G	G G
UL27	F: CATGGTTGGGTCCCCTGG R: CGCCAACTGATTGTATGGCA	50	186	0.4 [0.6]	2	TS (NS) TS (S)	T A	C G	C G	C G	C G	C G	T G	T G
UL36	F: AGATGTTTTAGTTCTGTGTGGGC R: GGCTGCACTCCTCCAAGATC	50	120	1.4 [1.3]	3	TS (NS) TS (S) TS (NS)	T T T	C C C	T T T	C C C	T T T	C C C	T T C	T T C
UL8	F: ATTGATGGAAGAGGAAATGAAGCA R: TTGTTTTCGCGGGTGTTAAGG	55	100	0.8 [1.2]	3	TS (NS) TV (NS) TV (NS)	T C C	C A A	T C C	C A A	T C C	C A A	T C A	T C A
IR	F: GTCAACGGGGCTAGATCATT R: ATTTACTTCTGGTTGCGTGCTC	50	100	0.6 [0.9]	2	TS (n/a) TV (n/a)	T T	C A	T T	T T	C A	C A	T T	T T
US4	F: TTCGTGTTCGGGGTGGATAG R: TCGAAATCAGGTATCCCGGC	54	125	0.5 [0.5]	3	TV (NS) TV (NS) TS (NS)	C T C	A G T	C T C	C T C	A G T	A G T	A G C	A G C
ORFB	F: CAGTGACGTTAATTTTACCGGGA R: CAGCTCGAGAAATTGCAGCG	50	71	0.6 [1.1]	1	TS (NS)	T	C	T	T	T	T	T	T
US7	F: ACTGGAAATCACGTCTCCGC R: GAATTGTAGCTTCGGGGCGA	50	76	1.5 [1.7]	3	TS (NS) TS (NS) TS (NS)	A A T	A G T	A A T	A A T	A G T	A G T	G G C	A G T

^a^ Predicted *T*_m_ gap values were calculated using uMelt Batch software

^b^*TS* transition, *TV* transversion, *S* synonymous, *NS* non-synonymous

**Table 3:** List of primers and selected target SNPs for the classification of US isolates

Target gene	Primer set (5’-3’)	Amplicon length	Predicted^a^*T*_m_ gap (°C)	No. of SNPs in amplicon	Type of SNP (NS/S)^b^	SNPs				
						Laryngo Vac	TCO IVAX	CEO TRVX	LT Blen	1874C5
UL54	F: TGTTCTGGCCGGGTCTATTG R: TTGTGTATCCGCGACCAGAG	62	1	1	TS (NS)	T	T	C	C	T
UL10	F: AAACCCTGCTTGCGGACTAA R: CTGGTGTTGATATCACTGGCCT	57	1.2	1	TS (NS)	T	C	T	T	C
ICP4-2	F: ATCGTCGTTGTCGGTCTTCC R: CACGTAGTAATGGACAGGCGA	69	0.9	1	TS (NS)	T	T	T	C	T
ICP4	F: CGGAGCACTTGCCGGTAC R: GTTGGCGGGAGATTCTTGGG	126	1.2	3	TS (S) TS (NS) TS (S)	T A T	T A T	T A T	T A T	C G C
						Expected strain-specific HRM combination pattern^c^				
						AAAA	ABAA	BAAA	BABA	ABAB

^a^ Predicted *T*_m_ gap values were calculated using uMelt Batch software

^b^*TS* transition, *TV* transversion, *S* synonymous, *NS* non-synonymous

^c^ In these 4-character HRM combination patterns, the letters represent the sequence type of UL54, UL10, ICP4 and a second region in ICP4 genes, respectively. The viruses with melting characteristics similar to those of the Laryngo Vac strain in each region are indicated by an “A” and otherwise by a “B”

For the HRM assay, all reactions were prepared using a QIAgility^®^ automated system (QIAGEN). Reaction mixtures (20 μL) were prepared containing 2 mM MgCl_2_, 200 μM each dNTP, 400 nM each forward and reverse primer, 8 μM SYTO^®^ 9 (Thermo Fisher Scientific), 0.04 U of GoTaq^®^ Flexi DNA Polymerase (Promega, Wisconsin, USA) per mL, Colorless GoTaq^®^ Flexi Buffer (Promega, Wisconsin, USA), and 2 μL of DNA template. PCR amplification and the HRM assay were performed on a Rotor-Gene Q 2plex HRM instrument (QIAGEN Inc., MD, USA) in 100-tube rings (Rotor-Disc 100; QIAGEN, Australia).

The thermal cycling profile included 45 cycles of a denaturation step at 95 °C for 30 seconds, an annealing step at a primer-specific temperature (listed in Table 2) for 30 seconds, and 30 seconds of extension at 72 °C. A final extension step at 72 °C for 5 minutes was included before the melting step. The amplified products were incubated at 95 °C for 2 minutes before a renaturation step at 60 °C for 2 minutes. The HRM was performed in the temperature range of 70-95 °C with fluorescence acquisition at every 0.3 °C and 2-second ramps. Every reaction was performed in triplicate and in three independent experiments unless stated otherwise. Controls in each run included a no-template control and the appropriate reference-virus-positive controls (Table 1). In order to generate a reference for identification of possible mixed populations that were not plaque purified as expected, a 1:1 mixture of the two parent strains was also included in the experimental setup to identify samples containing more than one variant type.

#### Principal component analysis of the HRM output

**Fig. 2:**
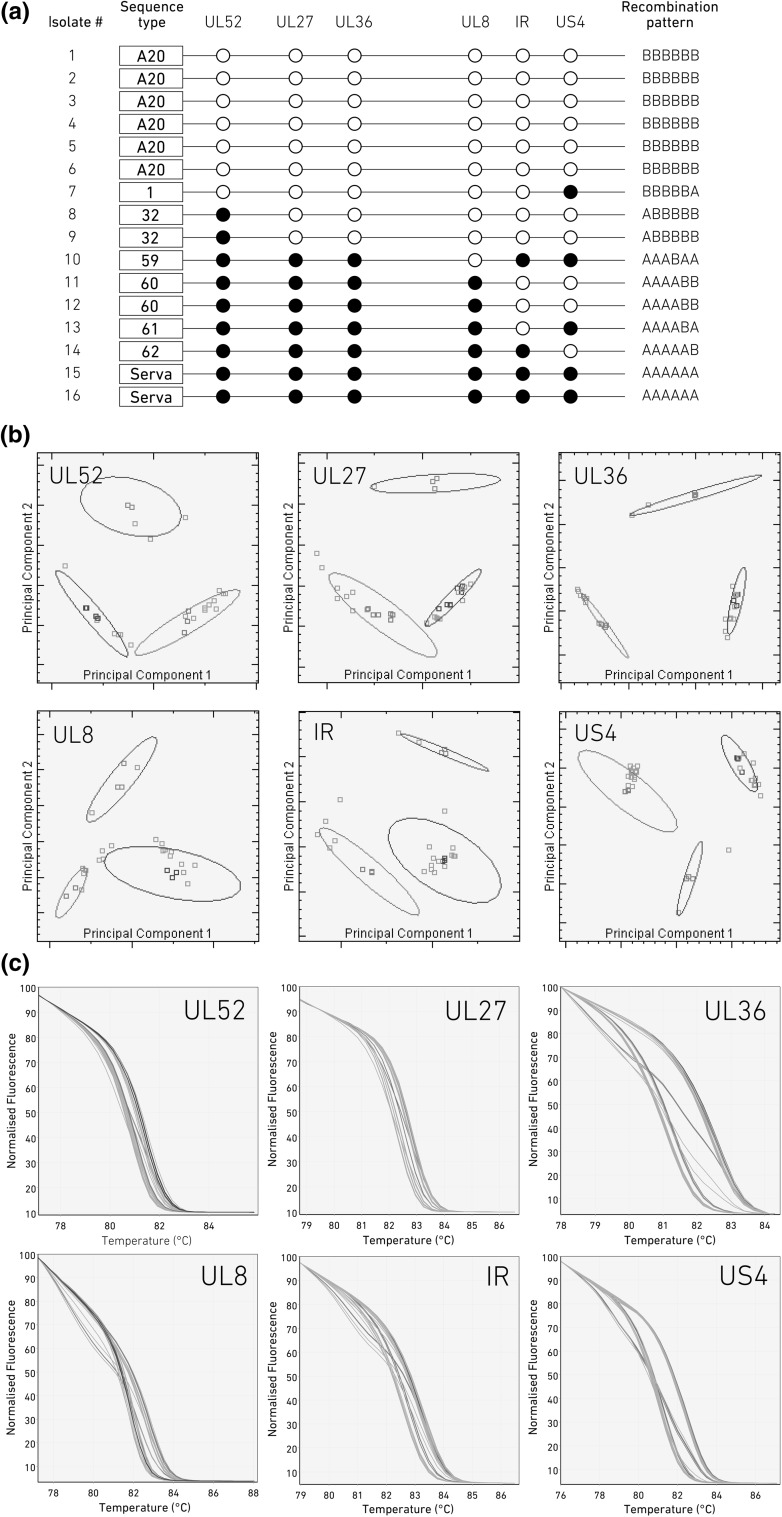
HRM analysis of parent and progeny viruses generated two days after infection of CEK cells with a 1:1 mixture of Serva and A20 ILTV. (a) A schematic representation of SNP patterns in parent and progeny viruses is shown, along with (b) PCA cluster analysis and (c) HRM plots of the assays for each of the six genomic regions. (a) A schematic view of genotypes of plaque-purified parent and progeny viruses after coinfection. The closed circles represent the Serva-like SNPs, and open circles indicate A20-like SNPs. Each virus is genotyped as A20, Serva, or recombinant with a unique genotype code number (1 to 62) for each recombination pattern. (b) PCA cluster plots and (c) HRM plots show triplicate Serva (red) and A20 (blue) ILTV parent strains as references. A 1:1 mixture of Serva and A20 ILTV (green) are shown to identify mixed samples. Plaque-purified progeny viruses (grey squares/curves) are clustered into Serva-origin (red oval) SNPs, A20-origin SNPs (blue oval) or mixed populations (green oval) on PCA plots. Clusters are highlighted by ovals that represent the covariance of the classified samples in a two-PC-dimension plot. The samples that plot outside the ovals are classified into the closest cluster and receive a lower typicality score

The HRM data were processed using Rotor-Gene Q Series software (version 2.1.0 Build 9) and Rotor-Gene ScreenClust HRM software (version 1.10.1.3) [29] in supervised mode. Principal component analysis (PCA) was used to better differentiate the HRM profiles based on the sequence variations in the parent viruses (A20 and Serva). The ScreenClust software normalizes the pre- and post-melting fluorescence levels, generates residual plots, identifies two or three principal components (PC) in the normalized data, defines clusters based on the control samples, assigns unknown/test samples into these defined clusters, and reports the probabilities and typicalities of unknown samples belonging to known clusters based on posterior class probabilities [29]. When all of the unknown samples are classified, the covariance of each cluster is determined (represented by ellipsoids in Fig. 2b). The mean of the reference samples forms the center of the cluster. The test samples are classified into each cluster with posterior probabilities and typicality scores (Supplementary Table 1). These were determined for pure populations of each of the parent viruses as well as for the control samples containing the 1:1 premixed template of each parent virus. The PCA plots of each parent and the mixed populations showed distinct and non-overlapping clusters that made it possible to clearly differentiate each population (each of the two parents and the mixed population). A range of recent studies have used the ScreenClust software for further analysis alongside traditional HRM analysis in higher-throughput settings [30–34].

#### *In silico* analysis for classification of published ILTV strains

The full genome sequences of five additional ILTV strains were collected from GenBank (Table 4) to enable *in silico* analyses and to examine the potential for a wider application of the HRM-based genotyping to ILTV strains, including strains from other geographical regions. Using the methods previously established with the Australian ILTV isolates, a series of *in silico* amplifications was performed in Geneious 11.1.4 [35] on multiple regions of five ILTV strains that originated from the USA (Table 3) in order to obtain strain-specific PCR products. Four new sets of primers (Table 3) were designed using Primer3 software (v. 2.3.4) [36]. The products of *in silico* PCRs were imported to uMelt Batch software (v. 2.2) [28] to predict the melting characteristics of each product.

**Table 4:** Viruses used in *in silico* classification of US isolates

Isolate	PCR-RFLP class [10, 37]	GenBank accession number	Reference
Laryngo Vac	IV	JQ083494	[38]
TCO IVAX	II	JN580312	[39]
CEO TRVX	IV	JN580313	[39]
LT Blen	IV	JQ083493	[38]
1874C5	VI	JN542533	[37]

## Results

### Differentiation of closely related ILTV genotypes by HRM analysis of SNPs

An HRM panel (Table 2) targeting SNPs across six sites (UL52, UL27, UL36, UL8, IR and US4) was applied to A20 and Serva vaccine strains and the well-characterized recombinant class 8, 9 and 10 ILTV strains (Table 1). A schematic view of the SNP pattern is shown in Figure 1A. The melt curve analysis from each of these reactions (Fig. 1B) on A20 and Serva was consistent with that predicted by the uMelt software, particularly in regards to the melting temperature separation predicted for each of the sites. This HRM analysis of six genomic regions enabled differentiation of each of the vaccine and recombinant field strains of ILTV (Fig. 1). The results obtained between assays were reproducible and showed 0.02 to 0.03% coefficients of variation between runs.

### Differentiation of parent from recombinant progeny viruses after recombination between Serva and A20 ILTV in coinfected primary cell cultures

The HRM panel targeting the same six sites (UL52, UL27, UL36, UL8, IR and US4) was applied to DNA extracted from plaque-purified viruses generated after a 1:1 coinfection of CEK monolayers with the A20 and Serva ILT vaccine strains (Fig. 2). Each tested region was classified as Serva-like or A20-like. Four isolates out of 20 tested samples were detected as mixed population of viruses and excluded from further analysis. The remaining 16 isolates were classified as either recombinant viruses or parent viruses (Fig. 2). In total, six different patterns of recombinants were detected, with no genotype(s) dominating amongst the recombinant progeny. Equal proportions of parent viruses and recombinant viruses were detected among the 20 progeny viruses tested (Fig. 2A).

### Adaptation of the HRM panel for genotyping a broader range of ILTV isolates

**Fig. 3:**
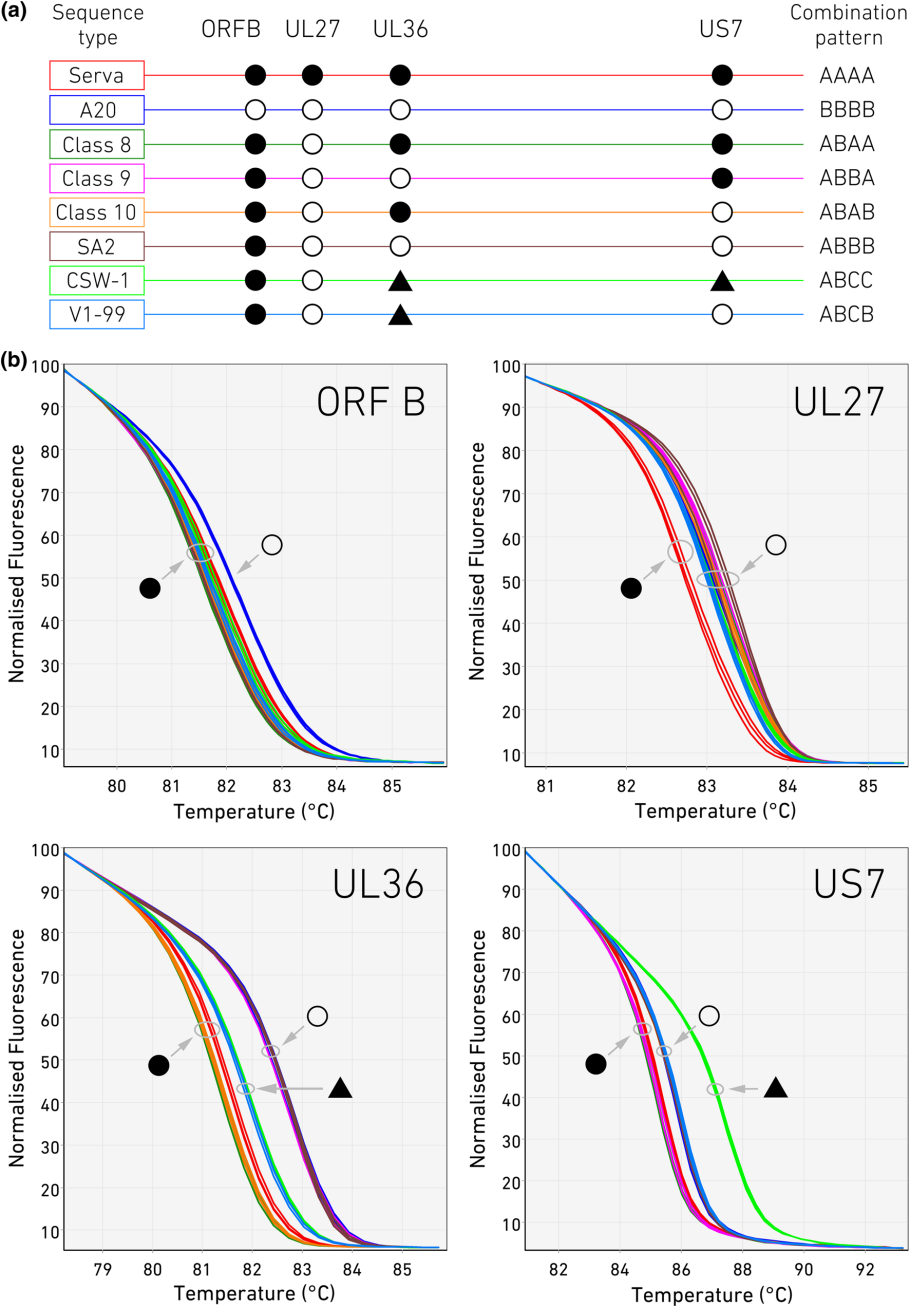
The genotyping of eight Australian ILTV field and vaccine isolates using HRM analysis. (a) A schematic view of a panel of four SNPs with distinct melting characteristics showing the distribution of Serva-like (closed circles), A20-like (open circles) and other SNPs (closed triangle) used to differentiate Australian field strains in Serva, A20, class 2, 4, 8, 9 and 10 ILTV strains and (b) HRM plots comparing four target genomic markers (ORFB, UL27, UL36 and US7) in a panel of viruses including Serva (red) and A20 (blue) to SA2 (brown), class 2 (V1-99) (light blue), class 4 (CSW-1) (light green), class 8 (dark green), class 9 (pink) and class 10 (orange) ILTV isolates. Each sample was clustered into either Serva-like (closed circles), A20-like (open circles) or other (triangles) SNP identities. The plots were generated by Rotor-Gene Q software. Each curve represents one replicate

Analysis of the six genomic regions across A20 and Serva genomes by HRM has shown the ability of this technology to build a picture of recombination across the ILTV genome, and this system was adapted to explore the potential for use in the genotypic classification of other ILTV isolates. To do so, the genomes of three more Australian strains (one vaccine [SA2] and two field strains [class 2 and 4]) were retrieved from GenBank and compared to the Australian ILTV isolates used previously in this study (Table 1). A panel of four SNPs was identified that would differentiate the three vaccine strains used in Australia (SA2, A20 and Serva) and five Australian genotypes (Table 1). This panel (Fig. 3A) includes two previously characterized regions (UL27 and UL36, Table 2, Fig. 1 and 2) and two new regions (ORFB and US7, Table 2, Fig. 3) predicted by uMelt to have a *T*_m_ gap of >1 °C. When this new panel of four sets of HRM primers (ORF B, UL27, UL36 and US7) was applied to extracted DNA from these viruses (Table 1), the actual HRM findings were used to generate a four-character combination code based on the melting characteristics of each of the target regions. Using these four sites, the genotype of the five distinct Australian field strains and the three vaccine strains used in Australia could be differentiated from each other (Fig. 3). Moreover, the SA2 and A20 vaccine strains could be differentiated from each other in one region (ORF B) despite their close relationship (sharing 99.9% nucleotide sequence identity) and both being classified by PCR-RFLP as class 1 viruses.

### *In silico* exploration of HRM-based genotyping to ILTV strains from other countries

Given the consistency between the predicted and experimentally observed melting temperatures for HRM analysis, this method could be broadened as a means of genotyping viruses from different geographical regions. To illustrate this, similar *in silico* analysis of five US strains (Table 4) has identified four regions that could be used to differentiate US field and vaccine isolates (Table 3), suggesting the potential for the simple application of this technology to characterize a broad range of ILTV isolates across the world.

## Discussion

In this study, we aimed to develop a reliable cost- and time-efficient method that can be applied to the classification and genotyping of ILTV strains and the identification of ILTV recombinant viruses. The majority of existing ILTV classification systems are based on identifying the sequence variations in representative regions of the genome that are first amplified by PCR. Sequence variation is identified in these regions by a range of methodologies including RFLP [9, 10], Sanger sequencing [7, 8] or fluorescently labelled TaqMan^®^ probes [14, 40, 41]. Both the number of regions being analyzed and the method of analysis will influence the precision of the classification and differentiation of ILTV isolates. Although PCR-RFLP results are relatively easy to interpret, other studies have suggested the use of Sanger sequencing of one, two [7, 42–45] or multiple [8] region(s) of ILTV genome as an alternative to RFLP.

The HRM analysis used in the current study could differentiate field and vaccine strains of ILTV and classify them into distinct genotypes that had previously been determined by PCR-RFLP [9] and full-genome sequencing [6, 18, 19]. Australian ILTV classes 3, 4, 5 and 6 were not examined in this study, as no genomic sequences were available for these viruses to validate the results. The results of HRM analysis were reproducible and were in accordance with other methods; however, the successful use of this method relies on the precision of techniques used during sample preparation, DNA isolation, amplification and melting, and post-reaction analysis steps, as described in detail by Slomka *et al.* [21]. In order to overcome the variations introduced by different isolation methods and sample composition, a single type of DNA isolation kit was used for both test samples and the samples used as reference strains in this study. The commercial vaccine strains were passaged once in CEK cells so that the composition of the reference strains and test samples would be similar. Automated systems were used for extraction and PCR preparation to maintain the uniformity of samples and reactions. The sizes of amplicons for each target region was also kept short (<200 bp) where possible in order to obtain a higher *T*_m_ gap between variant types. The uMelt melting curve prediction software provided a reliable guide for selecting target SNPs based on the melting peaks. The predicted melting temperatures were close to experimental melting peaks with only slight differences (zero to 0.4 °C) in some regions. Therefore, when designing the second and third set of primers, the regions with a *T*_m_ gap of ≥1 °C were preferred. In order to use this system to classify viruses submitted from outbreaks of disease in the field it is likely that viruses from any clinical samples would also require processing (isolation and DNA extraction) using uniform techniques. It is conceivable that future assays could be developed and optimized for direct use on specific clinical samples (e.g., tracheal tissue or conjunctival swabs) after extensive evaluations using DNA extracted directly from clinical specimens. This would have time and cost advantages and represents an important area of future work. The direct use of the HRM assay on clinical samples has been successfully applied to several pathogens in the past [46–49]. The potential of HRM in identifying mixed specimens has been reported to be primarily dependent on the quantity and proportion of the target DNAs in the mixture [50]. Current detection or genotyping methods for ILTV have not been able to rapidly identify the presence of mixed virus populations in laboratory or field samples. The potential of this method to identify mixed infections in these settings requires further exploration.

The HRM system developed in this study could also be used successfully to detect recombinant viral progeny. This represents the first time that HRM methods have been used to detect recombination in herpesviruses. Previous studies have shown that genomic recombination between Serva and A20 ILTV strains has occurred in the natural host and has resulted in field recombinants with higher levels of virulence [18]. The results of the current study showed that these two vaccine strains can recombine in coinfected (MOI = 10) CEK cells and generate a diverse pattern of recombinants. After this coinfection, recombinants accounted for 50% (8/16) of progeny viruses, and the highest proportion of an individual genotype (6/16) had an SNP profile identical to that of the A20 parent strain. The proportion of recombinants in the current study is consistent with the greater opportunity for coinfection of cells at this high MOI so that recombination can occur. These finding are consistent with findings from an *in vivo* coinfection study in SPF chickens using a mixture of CSW-1 and V1-99 ILTV strains delivered intratracheally, where 46.5% (in birds co-inoculated with 10^3^ PFU of each virus) to 79.2% (in birds co-inoculated with 10^4^ PFU of each virus) of isolated progeny viruses were identified as recombinants.

In conclusion, the HRM assay developed in this study was successfully applied as a high-throughput screening tool for recombinant detection and could be used to successfully classify different strains of ILTV. In these applications the assay was shown to be accurate, time-efficient and cost-efficient. The HRM genotyping method promises to be an important tool to facilitate the study of ILTV epidemiology and recombination in both experimental and field settings.

## Electronic supplementary material

Below is the link to the electronic supplementary material.

Supplementary material 1 (PDF 112 kb)
