# Supplementary material for: Development and application of high-resolution melting analysis for the classification of infectious laryngotracheitis virus strains and detection of recombinant progeny
Source: Arch Virol. 2018 Nov 12;164(2):427–38. doi: 10.1007/s00705-018-4086-1 (PMC6373279; doi:10.1007/s00705-018-4086-1)
Supplement: Supplementary file 1 — Supplementary material 1 (PDF 112 kb) [file 705_2018_4086_MOESM1_ESM.pdf]

Development and application of high resolution melting analysis for the classification of infectious laryngotracheitis viruses and detection of recombinant progeny

Archives of Virology

Omid Fakhri[1*], Carol A Hartley[1], Joanne M Devlin[1], Glenn F Browning[1], Amir H Noormohammadi[2], Sang-Won Lee[1,3]

[1] Asia-Pacific Centre for Animal Health, Faculty of Veterinary and Agricultural Sciences, The University of Melbourne, Parkville, Victoria, Australia

[2] Asia-Pacific Centre for Animal Health, Faculty of Veterinary and Agricultural Sciences, The University of Melbourne, Werribee, Victoria, Australia

[3] College of Veterinary Medicine, Konkuk University, Seoul, Republic of Korea

* Corresponding author: omid.fakhri@unimelb.edu.au

Supplementary Table 1: Probability and typicality scores for each tested strain/isolate. Generated by ScreenClust software.

					Probabilities[d]		
Reaction #	Sample label[a]	HRM Target region	Cluster[b]	Typicality[c]	Serva-like	A20-like	Mixed
1	Serva	UL52	Serva-like	0.874196	1.0000	0.0000	0.0000
2	Serva	UL52	Serva-like	0.435242	1.0000	0.0000	0.0000
3	Serva	UL52	Serva-like	0.602155	1.0000	0.0000	0.0000
4	A20	UL52	A20-like	0.986018	0.0000	1.0000	0.0000
5	A20	UL52	A20-like	0.822288	0.0000	1.0000	0.0000
6	A20	UL52	A20-like	0.982501	0.0000	1.0000	0.0000
7	Mixed std	UL52	Mixed	0.646321	0.0000	0.0000	1.0000
8	Mixed std	UL52	Mixed	0.646321	0.0000	0.0000	1.0000
9	P1	UL52	Serva-like	0.923305	1.0000	0.0000	0.0000
10	P2	UL52	A20-like	0.490563	0.0000	1.0000	0.0000
11	P3	UL52	Serva-like	0.216705	1.0000	0.0000	0.0000
12	P4	UL52	A20-like	0.265993	0.0000	1.0000	0.0000
13	P5	UL52	Serva-like	0.156532	1.0000	0.0000	0.0000
14	P6	UL52	Mixed	0.682577	0.0000	0.0000	1.0000
15	P7	UL52	A20-like	0.209699	0.0000	1.0000	0.0000
16	P8	UL52	Mixed	0.267606	0.0000	0.0000	1.0000
17	P9	UL52	Serva-like	0.749664	1.0000	0.0000	0.0000
18	P10	UL52	A20-like	0.031533	0.0000	1.0000	0.0000
19	P11	UL52	Serva-like	0.684698	1.0000	0.0000	0.0000
20	P12	UL52	Serva-like	0.45652	1.0000	0.0000	0.0000
21	P13	UL52	Serva-like	0.811807	1.0000	0.0000	0.0000
22	P14	UL52	Serva-like	0.331768	1.0000	0.0000	0.0000
23	P15	UL52	Serva-like	0.347001	1.0000	0.0000	0.0000
24	P16	UL52	Serva-like	0.042221	0.9590	0.0410	0.0000
25	P17	UL52	Serva-like	0.495575	1.0000	0.0000	0.0000

26	P18	UL52	A20-like	0.31982	0.0000	1.0000	0.0000
27	P19	UL52	A20-like	0.583388	0.0000	1.0000	0.0000
28	P20	UL52	Mixed	0.355839	0.0000	0.0000	1.0000
29	Serva	UL27	Serva-like	0.972047	1.0000	0.0000	0.0000
30	Serva	UL27	Serva-like	0.791332	1.0000	0.0000	0.0000
31	Serva	UL27	Serva-like	0.847056	1.0000	0.0000	0.0000
32	A20	UL27	A20-like	0.447492	0.0000	1.0000	0.0000
33	A20	UL27	A20-like	0.895133	0.0000	1.0000	0.0000
34	A20	UL27	A20-like	0.487201	0.0000	1.0000	0.0000
35	Mixed std	UL27	Mixed	0.572407	0.0000	0.0000	1.0000
36	Mixed std	UL27	Mixed	0.572407	0.0000	0.0000	1.0000
37	Mixed std	UL27	Mixed	0.572407	0.0000	0.0000	1.0000
38	P1	UL27	Serva-like	0.675542	1.0000	0.0000	0.0000
39	P2	UL27	A20-like	0.103159	0.0000	1.0000	0.0000
40	P3	UL27	A20-like	0.348297	0.0000	1.0000	0.0000
41	P4	UL27	A20-like	0.290435	0.0000	1.0000	0.0000
42	P5	UL27	Serva-like	0.241356	1.0000	0.0000	0.0000
43	P6	UL27	A20-like	0.74021	0.0000	1.0000	0.0000
44	P7	UL27	A20-like	0.066363	0.0000	1.0000	0.0000
45	P8	UL27	A20-like	0.40193	0.0000	1.0000	0.0000
46	P9	UL27	Serva-like	0.146722	1.0000	0.0000	0.0000
47	P10	UL27	A20-like	0.581781	0.0002	0.9998	0.0000
48	P11	UL27	Serva-like	0.195983	1.0000	0.0000	0.0000
49	P12	UL27	Serva-like	0.05165	1.0000	0.0000	0.0000
50	P13	UL27	Serva-like	0.750359	1.0000	0.0000	0.0000
51	P14	UL27	A20-like	0.290546	0.0000	1.0000	0.0000
52	P15	UL27	Serva-like	0.309781	1.0000	0.0000	0.0000
53	P16	UL27	A20-like	0.263484	0.0075	0.9925	0.0000
54	P17	UL27	Serva-like	0.393293	1.0000	0.0000	0.0000
55	P18	UL27	A20-like	0.454944	0.0000	1.0000	0.0000
56	P19	UL27	A20-like	0.674516	0.0000	1.0000	0.0000
57	P20	UL27	Mixed	0.391625	0.0000	0.0000	1.0000
58	Serva	UL36	Serva-like	0.391854	1.0000	0.0000	0.0000
59	Serva	UL36	Serva-like	0.991152	1.0000	0.0000	0.0000
60	Serva	UL36	Serva-like	0.24128	1.0000	0.0000	0.0000
61	A20	UL36	A20-like	0.863102	0.0000	1.0000	0.0000
62	A20	UL36	A20-like	0.836366	0.0000	1.0000	0.0000
63	A20	UL36	A20-like	0.991428	0.0000	1.0000	0.0000
64	Mixed std	UL36	Mixed	0.981401	0.0000	0.0000	1.0000
65	Mixed std	UL36	Mixed	0.665945	0.0000	0.0000	1.0000
66	Mixed std	UL36	Mixed	0.496606	0.0000	0.0000	1.0000
67	P1	UL36	Serva-like	0.409389	1.0000	0.0000	0.0000
68	P2	UL36	A20-like	0.165872	0.0000	1.0000	0.0000
69	P3	UL36	A20-like	0.711315	0.0000	1.0000	0.0000
70	P4	UL36	A20-like	0.730578	0.0000	1.0000	0.0000
71	P5	UL36	Serva-like	0.609391	1.0000	0.0000	0.0000
72	P6	UL36	A20-like	0.068476	0.0000	1.0000	0.0000
73	P7	UL36	A20-like	0.937196	0.0000	1.0000	0.0000
74	P8	UL36	Mixed	0.266283	0.0000	0.0000	1.0000
75	P9	UL36	Serva-like	0.386923	1.0000	0.0000	0.0000
76	P10	UL36	A20-like	0.168485	0.0000	1.0000	0.0000
77	P11	UL36	Serva-like	0.57955	1.0000	0.0000	0.0000
78	P12	UL36	Serva-like	0.546459	1.0000	0.0000	0.0000
79	P13	UL36	Serva-like	0.697461	1.0000	0.0000	0.0000
80	P14	UL36	A20-like	0.391483	0.0000	1.0000	0.0000
81	P15	UL36	Serva-like	0.131539	1.0000	0.0000	0.0000

82	P16	UL36	A20-like	0.198419	0.0000	1.0000	0.0000
83	P17	UL36	Serva-like	0.28274	1.0000	0.0000	0.0000
84	P18	UL36	A20-like	0.208802	0.0000	1.0000	0.0000
85	P19	UL36	A20-like	0.620022	0.0000	1.0000	0.0000
86	P20	UL36	Mixed	0.270988	0.0000	0.0000	1.0000
87	Serva	UL8	Serva-like	0.90979	1.0000	0.0000	0.0000
88	Serva	UL8	Serva-like	0.90979	1.0000	0.0000	0.0000
89	A20	UL8	A20-like	0.998374	0.0000	1.0000	0.0000
90	A20	UL8	A20-like	0.99121	0.0000	1.0000	0.0000
91	A20	UL8	A20-like	0.991341	0.0000	1.0000	0.0000
92	Mixed std	UL8	Mixed	0.517471	0.0000	0.0000	1.0000
93	Mixed std	UL8	Mixed	0.515335	0.0000	0.0000	1.0000
94	Mixed std	UL8	Mixed	0.772424	0.0000	0.0000	1.0000
95	P1	UL8	A20-like	0.250836	0.0001	0.9999	0.0000
96	P2	UL8	A20-like	0.529484	0.0000	1.0000	0.0000
97	P3	UL8	A20-like	0.733539	0.0000	1.0000	0.0000
98	P4	UL8	A20-like	0.880882	0.0000	1.0000	0.0000
99	P5	UL8	A20-like	0.272311	0.7835	0.2165	0.0000
100	P6	UL8	Mixed	0.403909	0.0000	0.0000	1.0000
101	P7	UL8	A20-like	0.365511	0.0000	1.0000	0.0000
102	P8	UL8	Mixed	0.333997	0.0000	0.0000	1.0000
103	P9	UL8	Serva-like	0.529324	0.9992	0.0008	0.0000
104	P10	UL8	A20-like	0.643233	0.0000	1.0000	0.0000
105	P11	UL8	A20-like	0.13947	0.0000	1.0000	0.0000
106	P12	UL8	A20-like	0.291954	0.0000	1.0000	0.0000
107	P13	UL8	Serva-like	0.547609	0.9997	0.0003	0.0000
108	P14	UL8	A20-like	0.260149	0.0000	1.0000	0.0000
109	P15	UL8	Serva-like	0.177541	0.9999	0.0001	0.0000
110	P16	UL8	A20-like	0.865039	0.0000	1.0000	0.0000
111	P17	UL8	A20-like	0.002252	0.0000	1.0000	0.0000
112	P18	UL8	A20-like	0.565292	0.0000	1.0000	0.0000
113	P19	UL8	A20-like	0.976643	0.0000	1.0000	0.0000
114	P20	UL8	Serva-like	0.198413	0.9979	0.0021	0.0000
115	Serva	IR	Serva-like	0.805854	1.0000	0.0000	0.0000
116	Serva	IR	Serva-like	0.9917	1.0000	0.0000	0.0000
117	Serva	IR	Serva-like	0.924779	1.0000	0.0000	0.0000
118	A20	IR	A20-like	0.981401	0.0000	1.0000	0.0000
119	A20	IR	A20-like	0.992494	0.0000	1.0000	0.0000
120	A20	IR	A20-like	0.999352	0.0000	1.0000	0.0000
121	Mixed std	IR	Mixed	0.612439	0.0000	0.0000	1.0000
122	Mixed std	IR	Mixed	0.448517	0.0000	0.0000	1.0000
123	Mixed std	IR	Mixed	0.666369	0.0000	0.0000	1.0000
124	P1	IR	Serva-like	0.570965	1.0000	0.0000	0.0000
125	P2	IR	A20-like	0.467544	0.0000	1.0000	0.0000
126	P3	IR	A20-like	0.990447	0.0000	1.0000	0.0000
127	P4	IR	A20-like	0.968079	0.0000	1.0000	0.0000
128	P5	IR	Serva-like	0.298821	1.0000	0.0000	0.0000
129	P6	IR	A20-like	0.007253	0.0000	1.0000	0.0000
130	P7	IR	A20-like	0.234166	0.0000	1.0000	0.0000
131	P8	IR	Mixed	0.501741	0.0000	0.0000	1.0000
132	P9	IR	Mixed	0.306662	0.0000	0.0000	1.0000
133	P10	IR	A20-like	0.833773	0.0000	1.0000	0.0000
134	P11	IR	A20-like	0.480631	0.0000	1.0000	0.0000
135	P12	IR	Serva-like	0.133808	1.0000	0.0000	0.0000
136	P13	IR	A20-like	0.702349	0.0000	1.0000	0.0000
137	P14	IR	A20-like	0.350133	0.0000	1.0000	0.0000

138	P15	IR	A20-like	0.623835	0.0000	1.0000	0.0000
139	P16	IR	A20-like	0.730398	0.0000	1.0000	0.0000
140	P17	IR	Serva-like	0.158997	1.0000	0.0000	0.0000
141	P18	IR	A20-like	0.71158	0.0000	1.0000	0.0000
142	P19	IR	A20-like	0.894707	0.0000	1.0000	0.0000
143	P20	IR	A20-like	0.001484	0.0000	1.0000	0.0000
144	Serva	US4	Serva-like	0.993494	1.0000	0.0000	0.0000
145	Serva	US4	Serva-like	0.994084	1.0000	0.0000	0.0000
146	Serva	US4	Serva-like	0.999797	1.0000	0.0000	0.0000
147	A20	US4	A20-like	0.835162	0.0000	1.0000	0.0000
148	A20	US4	A20-like	0.95849	0.0000	1.0000	0.0000
149	A20	US4	A20-like	0.982227	0.0000	1.0000	0.0000
150	Mixed std	US4	Mixed	0.572407	0.0000	0.0000	1.0000
151	Mixed std	US4	Mixed	0.572407	0.0000	0.0000	1.0000
152	Mixed std	US4	Mixed	0.572407	0.0000	0.0000	1.0000
153	P1	US4	Serva-like	0.311066	1.0000	0.0000	0.0000
154	P2	US4	A20-like	0.241988	0.0000	1.0000	0.0000
155	P3	US4	A20-like	0.63635	0.0000	1.0000	0.0000
156	P4	US4	A20-like	0.599478	0.0000	1.0000	0.0000
157	P5	US4	Serva-like	0.612067	1.0000	0.0000	0.0000
158	P6	US4	A20-like	0.745149	0.0000	1.0000	0.0000
159	P7	US4	A20-like	0.731094	0.0000	1.0000	0.0000
160	P8	US4	Mixed	0.565137	0.0000	0.0000	1.0000
161	P9	US4	Serva-like	0.991598	1.0000	0.0000	0.0000
162	P10	US4	A20-like	0.937948	0.0000	1.0000	0.0000
163	P11	US4	A20-like	0.81826	0.0000	1.0000	0.0000
164	P12	US4	A20-like	0.861941	0.0000	1.0000	0.0000
165	P13	US4	Serva-like	0.24958	1.0000	0.0000	0.0000
166	P14	US4	A20-like	0.751248	0.0000	1.0000	0.0000
167	P15	US4	A20-like	0.134296	0.0000	1.0000	0.0000
168	P16	US4	A20-like	0.334642	0.0000	1.0000	0.0000
169	P17	US4	Serva-like	0.192503	1.0000	0.0000	0.0000
170	P18	US4	A20-like	0.109779	0.0000	1.0000	0.0000
171	P19	US4	Serva-like	0.40864	1.0000	0.0000	0.0000
172	P20	US4	Mixed	0.000748	0.0000	0.0000	1.0000

[a] Known (control) samples were labelled as Serva, A20 or Mixed standard (1:1 mixture of Serva and A20). The viruses isolated from progeny population after co-infection were labelled P1 to P20.

[b] Samples are identified as either Serva-like, A20-like or Mixed SNP identity and clustered accordingly.

[c] The typicality measures how well a sample falls within the cluster distribution in which it has been classified.

[d] The probability of each sample fitting into a cluster. The samples are called into the cluster that has the highest probability.
